# Perceived barriers to the regionalization of adult critical care in the United States: a qualitative preliminary study

**DOI:** 10.1186/1472-6963-8-239

**Published:** 2008-11-17

**Authors:** Jeremy M Kahn, Rebecca J Asch, Theodore J Iwashyna, Gordon D Rubenfeld, Derek C Angus, David A Asch

**Affiliations:** 1 Division of Pulmonary, Allergy & Critical Care, University of Pennsylvania School of Medicine, 3400 Spruce Street, Philadelphia PA 19104, USA; 2 Leonard Davis Institute of Health Economics, University of Pennsylvania, 3641 Locust Walk, Philadelphia PA 19104, USA; 3 Center for Clinical Epidemiology & Biostatistics, University of Pennsylvania School of Medicine, 423 Guardian Drive, Philadelphia PA 19104, USA; 4 Division of Pulmonary and Critical Care Medicine, University of Michigan, 3A23 300 NIB, SPC 5419, 300 North Ingalls, Ann Arbor MI 48109, USA; 5 Program in Trauma, Emergency, and Critical Care, Sunnybrook Health Sciences Centre, University of Toronto, 2075 Bayview Avenue, Toronto ON, Canada M4N 3M5; 6 Department of Critical Care Medicine, CRISMA Laboratory, University of Pittsburgh School of Medicine, 3550 Terrace Street, Pittsburgh PA, USA; 7 Center for Health Equity Research and Promotion, Philadelphia Veterans Affairs Medical Center, 3900 Woodland Avenue, Philadelphia PA 19104, USA

## Abstract

**Background:**

Regionalization of adult critical care services may improve outcomes for critically ill patients. We sought to develop a framework for understanding clinician attitudes toward regionalization and potential barriers to developing a tiered, regionalized system of care in the United States.

**Methods:**

We performed a qualitative study using semi-structured interviews of critical care stakeholders in the United States, including physicians, nurses and hospital administrators. Stakeholders were identified from a stratified-random sample of United States general medical and surgical hospitals. Key barriers and potential solutions were identified by performing content analysis of the interview transcriptions.

**Results:**

We interviewed 30 stakeholders from 24 different hospitals, representing a broad range of hospital locations and sizes. Key barriers to regionalization included personal and economic strain on families, loss of autonomy on the part of referring physicians and hospitals, loss of revenue on the part of referring physicians and hospitals, the potential to worsen outcomes at small hospitals by limiting services, and the potential to overwhelm large hospitals. Improving communication between destination and source hospitals, provider education, instituting voluntary objective criteria to become a designated referral center, and mechanisms to feed back patients and revenue to source hospitals were identified as potential solutions to some of these barriers.

**Conclusion:**

Regionalization efforts will be met with significant conceptual and structural barriers. These data provide a foundation for future research and can be used to inform policy decisions regarding the design and implementation of a regionalized system of critical care.

## Background

The presence of a trained critical care physician and a multidisciplinary care team is associated with improved patient outcomes in the intensive care unit (ICU) [1-3]. Given the ortage of ICU clinicians, however, only a small minority of ICUs in the United States are organized in this manner [4]. The situation is expected to worsen as the population ages and demand for critical care rises [5]. To address this crisis some have called for a regionalized approach to critical care [6-8]. Under a regionalized scenario, high-risk patients would be routinely transferred to large regional care centers according to standardized triage criteria. Supporting this idea are several studies demonstrating lower risk-adjusted mortality in high volume hospitals and ICUs, indicating that the resources and experience of large hospitals may contribute to improved outcomes [9-12]. Recent population-based data indicate that transferring patients to high volume hospitals may significantly impact mortality for critically ill patients [13].

In order to research, design and implement a regionalized system of care, the active participation of all stakeholders will be essential. While there has been much written in support of regionalization by academics and opinion leaders, no information exists about the perceptions of front line critical care providers and hospital administrators regarding regionalization of care. Assessing the opinions of key stakeholders is a necessary step in implementing this type of large-scale organizational change [14]. Accordingly, the purpose of this study was to assess provider attitudes toward regionalization of adult critical care. We sought to determine not only the barriers to regionalization, but also potential solutions to those barriers. Because so little is known about the challenges to regionalization, including who would organize and implement such a system, we elected to use semi-structured interviews from a range of potential stakeholders in United States hospitals.

## Methods

### Study design and subjects

We performed a qualitative study using open-ended, semi-structured telephone interviews with potential stakeholders in critical care regionalization [15]. The interviews occurred between February and May, 2007. Stakeholders of interest included ICU physicians, ICU nurse managers, emergency department physicians, emergency department nurse managers, and hospital administrators/medical directors. These were identified as individuals who either routinely coordinate care for critically ill patients or may be involved in triage decisions under a regionalized scenario.

We obtained a stratified random sample of all non-federal, general care hospitals in the United States from the 2004 American Hospital Association Annual Survey. Hospitals were stratified by region (Northeast, Southeast, Midwest, and West), hospital size (<100 beds, 100 – 399 beds, and ≥400 beds) and size of metropolitan statistical area (<100,000, 100,000 – 1 million, and >1 million persons). The sampling frame was designed to ensure that we interviewed individuals representing a broad range of potential roles in a tiered, regionalized system of care. To account for the fact that many stakeholders either could not be reached or would decline to participate, three hospitals were selected from each strata. If fewer than three hospitals existed within a strata, all hospitals in the strata were selected.

### Interviews

The interview guide consisted of a series of open-ended questions about regionalization and a list of potential barriers and solutions. The guide was developed with input from local care-givers experienced in regional care delivery and focused on three domains: (a) knowledge of and attitudes toward regionalization, (b) barriers to regionalizing care, and (c) potential solutions to those barriers. In order to ensure the broadest possible range of responses and solicit important viewpoints on novel barriers and solutions as they were uncovered, the guide expanded and evolved over time as additional interviews were conducted.

At the beginning of each interview we explicitly defined regionalization by making the following statement: "In a regionalized system, all US hospitals would be categorized into tiers according to the level of ICU acuity they can provide. The criteria for each tier would be set by a multidisciplinary panel and monitored by existing accreditation organizations. Selected patients in lower level ICUs would then be routinely transferred to higher level, regional care centers."

Each hospital was called in random order, and hospital operators were used to identify potential stakeholders. We did not fix the total number of interviews in the study *a priori*. Rather, we conducted interviews until one of two pre-specified conditions was met: (a) we interviewed at least one of all five stakeholders in each strata of the sampling frame, or (b) we reached thematic saturation (i.e. no new barriers or solutions obtained in ten consecutive interviews). All participants gave oral informed consent. This research was approved by the University of Pennsylvania Institutional Review Board.

### Analysis

Interviews were recorded and transcribed. A framework for understanding regionalization was developed through directed content analysis of the interview responses [16]. Two investigators trained in qualitative data analysis (JMK, RJA) met regularly to identify key concepts and code the responses. Codes were tabulated, grouped into categories according to the developed framework, and reviewed with the other investigators [15]. Results are presented as a list of potential barriers and solutions, grouped by category, with representative quotes selected from the transcriptions used to illustrate each viewpoint [17].

## Results

The final sample contained 104 hospitals. Thematic saturation was achieved after conducting 30 stakeholder interviews at 24 hospitals. Hospitals with a participating stakeholder varied in size and location (Table 1). We interviewed eight hospital administrators (27 percent), seven ICU nurse managers (23 percent), five emergency department nurse managers (17 percent), seven ICU physicians (23 percent), and three emergency department physicians (10 percent).

**Table 1:** Characteristics of hospital in the general sample and hospitals at which a stakeholder participated in an interview.

**Variable**	**In sample (n = 104)**	**Participated (n = 24)**
***Region***		
Northeast	24 (23)	7 (29)
Southeast	29 (28)	3 (13)
Midwest	25(24)	6 (25)
West	26 (25)	8 (33)
		
***Community size***		
<100,000	32 (31)	4 (17)
100,000 – 1 million	36 (35)	11 (46)
> 1 million	36 (35)	9 (38)
		
***Total beds***		
< 100	36 (35)	5 (21)
100 – 399	36 (35)	8 (33)
≥ 400	32 (31)	11 (46)
		
***Critical care beds***		
Median [IQR]	14 [6–33]	28 [10–40]
Range	0 – 216	0 – 72
		
***University affiliated***	34 (33)	11 (46)
		
***Designated trauma center***	45 (43)	14 (58)

Values are presented as frequency (percents) unless otherwise noted.

IQR = interquartile range

Respondents had diverse opinions on how regionalization would affect patient care. Some respondents felt that regionalization would have a small impact on patient outcomes. This view was expressed by stakeholders who thought that critical care in their area was already regionalized under an *ad hoc *system ("In our community we actually function that way – our center is the destination spot for anyone who becomes critically ill beyond a certain level."), or those at hospitals that already receive a large number of referral patients ("we already have a high volume and state-of-the-art facility"). Respondents who felt regionalization would improve quality generally noted the concentration of resources for critically ill patients at high volume centers (" [patients] would be in a facility that would be able to offer the care that they needed, so would imagine their outcomes would be better"). Respondents also noted the potential for adverse outcomes, either by restricting patient market freedoms ("I think it would remove choice and negatively impact patient care"), by overcrowding large centers ("What do we do in the ER with our ICU patients?"), by reducing services at small-volume hospitals ("we would lose all of our skills and capabilities"), or through the direct risks of inter-hospital transfer ("if you are transferring someone who is critically ill there's always a risk").

Respondents expressed the idea that regionalization could potentially be very costly, noting that it would create the added expense of routine inter-hospital transfer, and that large hospitals would see an increase in complex cases. Respondents also noted ways in which regionalization could reduce the cost of care through economies of scale. Regional referral centers, which are predominantly staffed by intensivists, might provide complex care more efficiently ("you will have a higher level of expertise"). Concentrating critical services might also reduce duplication of services and increase efficiency through economies of scale (" [regionalization could] lower cost by focusing complex care in places that do it regularly"). Many stakeholders expressed uncertainty in this area, citing the need for cost-effectiveness studies prior to instituting a regionalized system. A few stakeholders framed the issue of costs in terms of reimbursement and net revenue for hospitals, noting that the overall financial impact is dependent on payer mix ("It depends on what we send – if we send no-pay patients, they'll lose money and we'll do okay".)

All respondents identified potential barriers to regionalization (Table 2). We categorized the reported barriers to regionalization into those concerning patients and families, clinicians, source hospitals, destination hospitals, and system design. Key concepts included financial and personal strain on families, lack of physician agreement to participate, the need for demonstrated effectiveness, and the possibility that regionalization would overwhelm the resources at destination hospitals. Respondents also noted the concern that regionalization could negatively impact resources and clinical skills at source hospitals, which may in turn lower the quality of care for patients left behind.

**Table 2:** Barriers to regionalization of adult critical care.

**Category**	**Sub-category**	**Illustrative quotes**
**Patient and Family**	Financial strain	*If you are asking someone to transfer a loved one four hours [away], and they choose to be with that loved one for weeks on end, there is an economic cost to that*.
		*The cost of having to drive 40–50 miles to a tertiary center could be a barrier*.
	Personal strain	*To get to a place only to end up getting to another place that's further away I think adds a lot of emotional stress to family*.
		*Nobody wants their loved one a hundred miles away, particularly when they're sick*.
**Physician**	General agreement	*I think it would be very difficult to get rural pulmonologists and critical care physicians to participate*.
		*I think it is better to cultivate some of the services locally*.
	Loss of income	*[They] stand to lose business. They don't want to lose the patients to the bigger hospitals*.
		*Everybody wants the payers; it's all money driven*.
	Loss of autonomy	*[Physicians will be] concerned that if they give up their patients they will never get them back*.
		*They like to take care of their patients no matter what*.
**Source hospital**	Loss of income	*If they took all of our sick patients it could be devastating to our facility*.
		*Patients equals volume equals financial viability*.
	Loss of care capacity	*If they took away all our sick patients, we would become a fairly useless institution*.
		*It will lower the variety of care a nurse is exposed to and therefore they will have less growth potential in technology*.
**Destination hospital**	Overwhelmed resources	*We're full all the time with what we've got*.
		*Nobody is equipped to handle that. We have trouble getting people in. Who's going to take those patients?*
		*Large facilities already divert patients regularly*.
	Cost and reimbursement	*Sometimes when you get the acutely ill in the ICU, they are coming with no insurance*.
		*Hospitals...have the potential for a great deal of financial woe, inheriting all the patients that might come to them for critical care without any reimbursement*.
**System**	Regulation	*Lack of authority to do any of this*.
		*I can't envision a central triage system that would work without controversy*.
	Cost and cost-effectiveness	*Cost is going to be a huge barrier*.
		*Need to find out whether the outcome justifies the expense*.
	Limited staffing	*We basically cannot use all of our beds because of our nursing situation*.
	Triage and infrastructure	*The infrastructure is not there to do this*.
		*It would be hard to make the call quickly regarding where the patient should go*.
		*There would have to be additional [ambulance] rigs put into play so we're not missing the 911 calls*.

Potential solutions to these barriers were grouped into four domains: communication, education, system design, and reimbursement (Table 3). Key concepts included maintaining continuity of care, developing an evidence base, and educating clinicians. Some stakeholders conceived of a system where all the hospitals in a regional referral hospital shared a common information technology platform, which would facilitate communication and allow rapid transfer of health information between sites. Many stakeholders indicated that the criteria to become a regional referral center should be internal and voluntary – i.e., any hospital should have the potential to become a high level critical care center by meeting a set of objective criteria. Stakeholders also felt that a regionalized system should allow for some return of reimbursement and ultimately patient care to the source hospitals as a way to overcome losses of patients and revenue.

**Table 3:** Potential strategies to overcome barriers to regionalization of adult critical care.

**Category**	**Sub-category**	**Illustrative quotes**
**Communication**	Continuity of care	*I don't think it breaks continuity, as long as the destination facility is in regular communication with the referring facility and communication*.
		*You need communication back and forth – standardized forms or processes between source and destination that everyone would use*.
	Feedback	*I think creating a mechanism for timely feedback of the care of those patients will be important*.
	Information Technology	*Centralized call center for all potential referrals to the main hospital*.
		*Install the same software program between hospitals so that all records can be transferred electronically. Then you can push the records back and forth between facilities*.
**Education**	Develop and disseminate evidence of benefit	*Education of families and of physicians of the need to do this and the benefits to this would be a tremendous asset*.
**System design**	Tier designation based on voluntary service level	*It would have to be a mandated criteria for keeping patients at certain seriousness of illness, in order to support a facility that would pass some agency's inspection*.
		*One solution would be to regionalize different strengths, if for instance, our hospital kept cardiac and CCU capabilities, and sent stroke patients to the other hospital*.
	Broad stakeholder participation	*EVERYONE would have to be involved*.
	Objective triage criteria	*Have criteria for what would warrant the patients being transferred. Therefore the doctor wouldn't feel like they failed*.
**Reimbursement**	Feedback revenue to referring centers	*Reimbursement rates, so that the hospitals that are doing the transferring can have some reimbursement for the care of those patients*.
		*Reimburse the sending hospital for at least a portion of the care*.
	Transfer patients back to source hospitals after improvement	*The patient is appropriately channeled back with the appropriate documentation*.
		*If people need to have acute rehab, it should be back at the primary unit where the family support is*.

## Discussion

This study demonstrates that front-line critical care stakeholders in the United States hold diverse opinions about the benefit of regionalization and potential barriers to implementing a regionalized system. Many stakeholders felt that regionalization had the potential to significantly improve outcomes for critically ill patients in small hospitals, while others expressed concerns that regionalization could negatively impact care for some patients. Costs, personal and family strain, loss of revenue and loss of autonomy were seen as important barriers to implementing a regionalized system. Potential solutions to these barriers included improving communication between hospitals, developing common information technology platforms, and revenue sharing between hospitals within a system.

The framework developed in this study provides an foundation for future research and development of a regionalized system of critical care. Formal regionalization of adult critical care in the United States has been under study since at least 1994, when a taskforce of the American College of Critical Care concluded that regionalizing critical care is likely to improve patient outcomes and called for more research in the area [6]. More recently, a position paper of the critical care professional societies highlighted regionalization as a potentially important way expand access to intensivist-led, multidisciplinary care [7]. This report led to the Prioritizing the Organization and Management of Intensive Care Services (PrOMIS) conference, a multi-disciplinary taskforce consisting of critical care practitioners, non-critical care clinicians, payer groups, government and regulatory agencies, and patients [8]. PrOMIS participants concluded that variation in the delivery of critical care results in avoidable mortality and morbidity, and directly called for the creation of a tiered, regionalized system of care in the United States. Ultimately, the PrOMIS conference will lead to continued discussions about the regionalization of critical care, however formal regionalization efforts on a national scale are not yet underway. Much health policy in the United States is formed by subtle government regulation of open market processes, and it is likely that regionalization will proceed in this manner as well.

The present study adds to the regionalization debate by providing the views of a broader constituency of clinicians and administrators. Whereas opinion-leaders and researchers appear to be very supportive of regionalization, there is no such consensus among providers on the ground.

Many stakeholders worried that regionalization could disrupt existing patient-clinician relationships, create undue personal and financial hardships on families, limit the ability of small hospitals to provide necessary acute care, reduce revenue to small hospitals, and burden large hospitals with excess case-load. Still, other stakeholders expressed the core concept in support of regionalization: the notion that concentrating limited resources in a few selected large hospitals could improve both quality and efficiency for patients with critical illness.

Stakeholders also identified several key unknowns that can define a research agenda to be addressed prior to implementation. Many were unwilling to consider implementing a regionalized system without additional investigations into the effectiveness of such a system. Data are also needed as to how regionalization would affect case-load at referral hospitals. Even at large hospitals, existing ICU staffing and infrastructure are limited [18-20]. It is possible that the number of patients transferred to large hospital would overload existing resources. Similarly, we do not know how regionalization would affect small hospitals, where there is concern that regionalization might reduce their ability to care for acutely ill patients. Critical care is tightly tied to other hospital services such as cardiac, cancer and neurological care [21-24]. If hospitals are asked to scale back critical care services, they might need to scale back these services as well, potentially resulting in significant revenue loss and loss of community access to care. Finally, stakeholders expressed concern about the personal and financial hardships patients and families might face in travel to regional centers.

Stakeholders also suggested ways to overcome the barriers they perceive. To overcome financial barriers, it might be possible to return revenue to source hospitals. To overcome concerns about loss of continuity, information systems could be used facilitate communication across care sites. To address concerns about hospital and physician autonomy, all hospitals could be invited to become regional referral centers based on pre-defined objective criteria. Although some very small hospitals might not plausibly be able to compete for participation, hospitals are much more likely to participate if every hospital is given the opportunity to fulfill the criteria to accept high acuity patients in transfer. Indeed, this type of participation scheme was integral to trauma system development [25].

We interviewed only critical care stakeholders in the United States – these results may not directly apply to other countries with different approaches to the organization and management of critical care [26]. Countries with single-payor health systems like Canada and the United Kingdom may experience unique challenges should they attempt to regionalize critical care. Unlike the United States, closed ICUs staffed by trained intensivists are extremely common. Although research demonstrates volume-outcome relationships exist in Europe just as in the United States, the potential benefits may be lessened in the setting of universal intensivist staffing [12].

Our study has several additional limitations. First, we did not interview all potential stakeholders, such as respiratory therapists, pharmacists, primary care physicians, surgeons, or representatives from payer groups and regulatory agencies. We did not interview surgeons because regionalization for high-risk surgery is currently being advocated separately from critical care [27]. Others were not interviewed because we wanted to interview only those individuals who would be directly involved in the triage of critically ill patients under a regionalized system. The goal of this study was to give voice to those immediate, but otherwise under-represented, stakeholders. Second, our study was limited to stakeholders' perceptions of the barriers to regionalization. This study design may not reveal some important types of barriers, for example, socioeconomic, racial or professional status barriers which may be less well perceived by stakeholders or may be less socially acceptable to reveal. Nonetheless, an understanding of the perceptions of key stakeholders is an essential first step toward implementing a major organizational change such as regionalization. Third, we performed only 30 interviews, creating the possibility that some viewpoints were missed. We stopped after 30 interviews because we reached thematic saturation – additional interviews would be very unlikely to solicit new viewpoints. Fourth, stakeholder opinions are likely to vary across different regions and health systems – efforts to design a regionalized system based upon this research should be customized for local health care environments. Finally, although we sought to identify opinions and barriers to regionalization, we did not seek to quantify their absolute or relative importance, or determine the differences in perception of regionalization across stakeholder groups. These analyses are better suited to quantitative research methods. Instead, we took advantage of qualitative methodology to solicit the broadest possible viewpoints on regionalization. Future work should be directed at a quantitative examination of the perceptions of critical care stakeholders using survey methodology based upon these findings [28].

## Conclusion

Using open-ended interviews with key stakeholders, we have identified diverse opinions about the potential benefits of regionalization and the barriers to implementing a regionalized system. These results provide a roadmap for future research toward a more effective system of critical care.

## Competing interests

The authors declare that they have no competing interests.

## Authors' contributions

JMK contributed to study conception and design, analyzed the qualitative data, interpreted the results, and drafted the manuscript. RJA performed and transcribed the interviews, analyzed the qualitative data, and critically revised the manuscript for important content. TJI contributed to study conception and design, interpreted the results, and critically revised the manuscript for important content. GDR contributed to study conception and design, interpreted the results, and critically revised the manuscript for important content. DCA contributed to study conception and design, interpreted the results, and critically revised the manuscript for important content. DAA contributed to study conception and design, interpreted the results, and critically revised the manuscript for important content.

## Pre-publication history

The pre-publication history for this paper can be accessed here:


